# Viral Population Heterogeneity and Fluctuating Mutational Pattern during a Persistent SARS-CoV-2 Infection in an Immunocompromised Patient

**DOI:** 10.3390/v15020291

**Published:** 2023-01-19

**Authors:** Martina Brandolini, Silvia Zannoli, Giulia Gatti, Valentina Arfilli, Monica Cricca, Giorgio Dirani, Agnese Denicolò, Simona Semprini, Laura Grumiro, Manuela Imola, Damiano Larne, Maria Michela Marino, Martina Manera, Andrea Mancini, Francesca Taddei, Manuel Zagarrigo, Carlo Biagetti, Vittorio Sambri

**Affiliations:** 1 Unit of Microbiology, The Greater Romagna Area Hub Laboratory, 47522 Cesena, Italy; 2 Department of Experimental, Diagnostic and Specialty Medicine (DIMES)—Alma Mater Studiorum, University of Bologna, 40138 Bologna, Italy; 3 Unit of Ematology, AUSL Romagna, 47900 Rimini, Italy; 4 Unit of Infectious Diseases, AUSL Romagna, 47900 Rimini, Italy; 5 Department of Medical and Surgical Sciences (DIMEC)—Alma Mater Studiorum, University of Bologna, 40138 Bologna, Italy

**Keywords:** SARS-CoV-2, COVID-19, immunocompromised patients, intra-host evolution, NGS whole-genome sequencing

## Abstract

Literature offers plenty of cases of immunocompromised patients, who develop chronic and severe SARS-CoV-2 infections. The aim of this study is to provide further insight into SARS-CoV-2 evolutionary dynamic taking into exam a subject suffering from follicular lymphoma, who developed a persistent infection for over 7 months. Eight nasopharyngeal swabs were obtained, and were analyses by qRT-PCR for diagnostic purposes. All of them were considered eligible (Ct < 30) for NGS sequencing. Sequence analysis showed that all sequences matched the B.1.617.2 AY.122 lineage, but they differed by few mutations identifying three genetically similar subpopulations, which evolved during the course of infection, demonstrating that prolonged replication is paralleled with intra-host virus evolution. These evidences support the hypothesis that SARS-CoV-2 adaptive capacities are able to shape a heterogeneous viral population in the context of immunocompromised patients. Spill-over of viral variants with enhanced transmissibility or immune escape capacities from these subjects is plausible.

## 1. Introduction

Since its emergence in late 2019, severe acute respiratory syndrome coronavirus 2 (SARS-CoV-2), has proven an unprecedented adaptive capacity, which primarily allowed its almost uncontrolled human-to-human transmission and its intercontinental spread [1,2,3]. Although possessing a relatively lower genomic variability when compared to other RNA viruses, due to the proofreading activity exerted by non-structural protein 14 (or ExoN) [4], SARS-CoV-2 evolution has by far shown no sign of being limited, as demonstrated by the periodic appearance of new variants, either classified as VOI (Variants Of Interest) or VOC (Variants Of Concern), the latter bearing mutations that can increase viral infectivity, reduce effectiveness of diagnostics and therapeutics or contribute to the evasion from antibody immune response, both developed following a previous infection as much as induced by vaccination, thus potentially paving the way for SARS-CoV-2 continuing circulation rather than extinction [5,6]. As of the causative forces behind SARS-CoV-2 evolution, little space has remained for speculations, as more and more studies point at the significance of endogenous immune response or exogenous antibodies administration (either monoclonal antibodies or convalescent plasma derivatives) in shaping genomic diversity and pushing viral evolution forward. These comprehend both in vitro studies, conducted by culturing virus to monoclonal antibody [7] or polyclonal sera [8] selective pressure or by monitoring virus evolution within immunocompromised patients with a diminished or abolished immune response, often compensated by immunological treatments (convalescent plasma or monoclonal antibodies). In this perspective, these studies have often highlighted how infection in immunosuppressed patients with haematological malignancies treated with B-cell-depleting therapies may lead to persistent and uncontrolled viral replication as the most recognized sequela of acute infection. Typical of this type of infections is a particular intra-host viral evolution characterized by the accumulation of an unusually high number of mutations potentially relevant both from a biological and epidemiological point of view [9,10,11,12,13]. 

In this study, we present a case of a deeply immunocompromised patient suffering from stage III-A Follicular Lymphoma (BCL2+) and underlying *Cytomegalovirus* and *Pneumocystis jirovencii* infections, who, although being double-vaccinated (Comirnaty BNT162b2, BionTech/Pfizer), developed a severe SARS-CoV-2 infection, which resulted in prolonged (over 7 months at the time of writing) high-load viral shedding. By monitoring viral mutations accumulated over time, we managed to characterise peculiar intra-host viral evolutionary dynamics, thus demonstrating that sustained high-level replication can be coupled with viral evolution and consequently population diversification. Sequencing data were coupled with periodical serological screening, in order to define a correlation between immune response parameters and viral evolution. The obtained data suggested that within-host selection may parallel evolutionary dynamics on larger scales, i.e., on the population level, making immunocompromised individuals potential reservoirs of antigenically novel variants hinting at the speculation that they represent an index population for viral variants evolution and spread [14]. 

## 2. Materials and Methods

### 2.1. Clinical Case Presentation 

A 61-year-old male patient was diagnosed in February 2018 with stage III-A Follicular Lymphoma (BCL2+), was treated with Rituximab-Bendamustine and then with Rituximab as maintenance therapy. In March 2021 he was diagnosed with a *Cytomegalovirus* (CMV) infection, successfully treated with Valganciclovir. He received two doses of Comirnaty (BNT162b2) BionTech/Pfizer Vaccine, the last at the beginning of October 2021. He first tested positive for SARS-CoV-2 by qRT-PCR (Quantitative Reverse-Transcription Polymerase Chain Reaction) on 3 December 2021 and on December 7 was treated with Bamlanivimab/Etesevimab (LY-CoV555/LY-CoV016) monoclonal antibodies. On December 24 he was hospitalised for interstitial pneumonia and was treated with methylprednisolone. He ultimately tested negative for SARS-CoV-2 on December 28 and was discharged from the hospital. On February 4, again, he tested positive for SARS-CoV-2 and computed tomography (CT) scans and thoracic radiographies were performed, revealing marked interstitial-alveolar alterations and ground glass regions. From February 28 he was treated with Paxlovid for five days, with an initial near complete resolution of the symptomatology and to a good recovery of the respiratory function, but he was soon readmitted to the hospital with mild respiratory insufficiency. In March an overlapping opportunistic infection caused by *Pneumocystis jirovencii* was diagnosed and treated with Atovaquone, followed by lymphocyte typing (CD4+ = 224/µL, CD8+ = 715/µL, CD4+/CD8+ = 0.31) and HIV test, the latter resulting negative. Bone marrow biopsy was carried out on May 18: it did not show a progression of the haematological disease, nonetheless highlighting a considerable B-cell depletion and a low CD4+/CD8+ cell ratio (0.31, CD4+ = 173/µL, CD8+ = 560/µL). On 31 May intravenous administration of human normal immunoglobulin was started as supportive therapy for severe hypogammaglobulinemia (total serum IgG levels < 200 mg/dL); administration was repeated every 21 days. 

### 2.2. Molecular and Serological Diagnostics 

Nasopharyngeal swabs were collected from the patient between 3 December 2021 and 9 July 2022 as part of his routine clinical care for viral load monitoring. During the considered period, nasopharyngeal swabs were persistently positive for SARS-CoV-2 by qRT-PCR (quantitative reverse-transcription polymerase chain reaction), except for the one taken on 28 December 2021, which came back negative. All samples were routinely tested using Xpert Xpress SARS-CoV-2 (Cepheid, Sunnyvale, CA, USA) [15]. For every sample, N gene cycle threshold (Ct) was used as an approximation for viral load, which was rather high and did not significantly change over time, highlighting the impossibility for the patient’s immune system to counteract viral replication at any timepoint throughout the infection. Ct values from routine molecular testing are reported in Table 1. 

Starting from 27 December 2021, periodical serological tests were performed in order to monitor anti-Spike and anti-Nucleocapsid specific antibodies (IgG). Anti-Spike antibodies, measured with a commercial CLIA-based kit, LIAISON SARS-CoV-2 trimeric-S (DiaSorin, Vicenza, Italy) [16] peaked in late December 2021, following monoclonal antibody administration, and later steadily but consistently decreased until reaching a plateau between May and June 2022 (from Day 152 sample). Anti-Nucleocapsid antibodies, measured with a commercial CMIA-based kit, Abbott SARS-CoV-2 anti-NP IgG (Abbott Core Laboratory Systems, Lake Forest, IL, USA) [17] were undetectable at any timepoint, suggesting the absence of an endogenous immune response and the presence of passive immunity following the administration of monoclonal antibodies, which waned over time. IgG titres for anti-S and anti-N antibodies obtained from routine serological testing are reported in Table 2. 

### 2.3. Clinical Samples Inclusion for Longitudinal Evolution Monitoring

In total, eight clinical samples collected between April 14 (132 days after the first positive test) and 9 July 2022 (218 days after the first positive test) were included in the study. We became aware of the prolonged infection after about four months; we therefore could not include the samples collected prior to April 14 as they were discarded by the laboratory after being tested for diagnostic purposes. Before being included in this study, the sample underwent an anonymization procedure, in order to adhere to the regulations issued by the local Ethical Board (AVR-PPC P09, rev.2; based on Burnett et al., 2007 [18]). As previously mentioned, all samples were tested for diagnostic purposes with Xpert Xpress SARS-CoV-2 (Cepheid, Sunnyvale, CA, USA). Eligibility for subsequent sequencing was determined based on N gene Ct values. 

#### 2.3.1. Viral RNA Extraction, Library Preparation and Sequencing

Considering the high viral load of the samples, approximately inferred from qRT-PCR data, all samples were considered eligible for sequencing, as they had a relatively high viral load (Ct < 30, considered as the lower limit to obtain a reliable genomic sequence). Semi-quantitative estimation of viral load in swabs was assessed based on N gene cycle threshold (Ct) values.

After RNA extraction and purification performed using the Maelstrom 9600 system (TANBead—Taiwan Advanced Nanotech Inc., Taiwan), library preparation was performed using the CleanPlex SARS-CoV-2 Flex Research and Surveillance NGS Panel (Paragon Genomics, Inc., Hayward, CA, USA). All the protocol steps (viral RNA reverse transcription, multiplex PCR, digestions and indexing PCR) were performed according to the manufacturer’s instructions using the two-pool workflow for multiplex amplification and i7 and i5 indexes for Illumina for final indexing [19]. The entire process was performed using the Microlab STAR automated workstation (Hamilton, Reno, NV, USA). Libraries were then quantified using a Qubit Fluorometer with the dsDNA HS Assay Kit (Thermo Fisher Scientific, Waltham, MA, USA), normalized to 10 nM, pooled in equimolar ratios to reach the recommended final concentration of 4 nM following the Standard Normalization protocol on MiSeq System Denature and Dilute Libraries Guide (Illumina, San Diego, CA, USA), and finally denatured and diluted to 1 pM. Paired-end and dual-indexed sequencing was carried out on a MiSeq instrument (Illumina), with reagent kit v2, using 5% of 10 pM spike-in PhiX as control for low diversity libraries Paired-end, dual-indexed sequencing was carried out on a MiSeq instrument (Illumina Inc., San Diego, CA, USA). 

#### 2.3.2. Data Analysis 

Sequenced reads were aligned and compared with the reference genomic sequence of SARS-CoV-2 Wuhan-Hu-1 isolate (Access: NC_045512, Version: NC_045512.2) using SOPHiA-DDM-v4 (SOPHiA Genetics, Lausanne, Switzerland), for determination of the consensus sequence and variant calling, considering a 70% frequency cut-off threshold. Lineage assignment was performed using Phylogenetic Assignment of Named Global Outbreak LINeages (Pangolin) [20]. Clinical isolates sequences were hence compared with a reference B.1.617.2 AY.122 sequence (Access: OW998398.1) to determine whether the identified mutations were B.1.617.2 AY.122 lineage-defining mutations or rather derived from within-host viral evolution

## 3. Results

**Figure 1:**
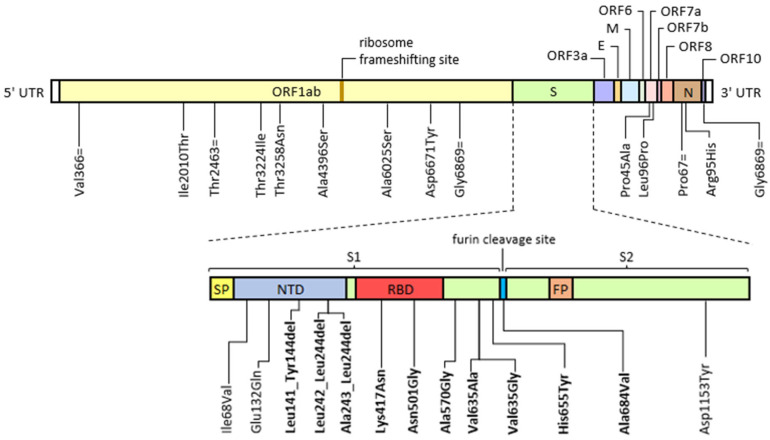
Graphical representation of permanently acquired and temporarily emerged mutations identified by deep-generation sequencing (Illumina) during the course of a prolonged infection in an immunocompromised patient compared to a reference B.1.617.2 AY.122 consensus sequence (Access: OW998398.1). Mutations falling on the spike coding sequence were shown separately in order to highlight the specific functional domain involved. Abbreviations: E, envelope; FP, fusion peptide; M, membrane; N, nucleocapsid; NTD, N-terminal domain; NP, nasopharyngeal swab; ORF, open reading frame; RBD, receptor binding domain; S, spike; S1 and S2, sub-unit 1 and sub-unit 2; SP, signal peptide; UTR, un-translated region. The “=” symbol indicates a synonym mutation, while “del” indicates a nucleotide deletion. Known escape and infectivity-enhancing mutations are highlighted in bold.

All genomic sequences matched the B.1.617.2 AY.122 lineage, which was broadly circulating in Italy until the end of December 2021. Sequence data analysis revealed the presence of a structured and complex as well as dynamic viral population. A chronology of persistent and temporary newly identified mutations is reported in Figure 1 and Table 3. 

In general, considering an estimated mutation rate of 6·10^−4^ mutations/genome/year (CI: 4 × 10^−4^–7 × 10^−4^) [21,22], the calculated mutation rate in this case, given all the mutational events occurred between Day 132 and Day 218 (86 days span), is 3.5 × 10^−5^, significantly higher than expected, hinting at an accelerated intra-host viral evolution. Taking into account the entire period (218 days), the resulting mutation rate is 2 × 10^−5^, still considerably high, although possibly underestimated due to the lack of information regarding the evolutionary events occurred in the first 132 days of infection. 

Overall, we were able to detect three genetically distinct subpopulations, which presumably coexisted throughout the infection, but differently emerged during the considered time period, hence modifying the relative composition of the viral population. In general, from day 132 to day 176, we noticed an interchange between two distinct but related subpopulations, which alternatively expanded and contracted over time. The first subpopulation comprehends Day 132, Day 152 and Day 176 swabs, characterised by 14 to 15 sequence variants (8 missense mutations, 3 synonym mutations, 2 to 3 deletions and 1 non-coding mutation) compared to a reference B.1.617.2 AY.122 consensus sequence. On the other hand, Day 144 and Day 165 swabs define the second subpopulation, with 15 sequence variants (8 missense mutations, 4 synonym mutations, 2 deletions and 1 non-coding mutation). In total, the two subpopulations have 13 mutations in common, which were persistent throughout the infection, while 4 other mutations appeared temporarily in one population or another, fluctuating over time and being replaced either by the wild-type or by another mutation. The late phase of the infection, was in turn dominated by a third subpopulation, presumably derived from the evolution of subpopulation 2, as they share 15 mutations, 13 of which were also maintained in both subpopulations 1 and 2, while 2 were peculiar of the second subpopulation. This population bears a greater number of mutations: 20 in Day 189 and Day 200 samples (13 missense mutations, 4 synonym mutations, 2 deletions and 1 non-coding mutation) and 26 in Day 218 sample (19 missense mutations, 4 synonym mutations, 2 deletions and 1 non-coding mutation). 

The mutations identified in this study involved, for the most part, the S gene (14 of 28 sites, 50%), but also those involving ORF1ab are conspicuous (9 of 28 sites, 32%). Many of these were identified in immunocompromised patients or induced in cell culture by exposure to monoclonal antibodies or neutralising sera (please refer to the literature cited in the introduction). In particular, evolved S gene sequence contains internal deletions on the Spike N-Terminal Domain (NTD), falling within the Recurrent Deletion Regions (RDR) 2 and 4 [10,23]. These include: Leu141_Tyr144del, Leu242_Leu244del and Ala243_Leu244del. All these deletions have been demonstrated to disrupt major immunodominant epitopes recognized by the neutralizing antibody response, hence conferring an augmented es-cape potential. The epitope including the amino acids 141-144 is also mutated in the B.1.1.7 and B.1.525 lineages (Tyr145del) and in the B.1.1.529 BA.1 lineage (Gly142_Tyr145delinsAsp). Another minor neutralization escape variant is represented by Val635Ala [24]. In the later phase, amino acid at position 635 is in turn substituted by a glycine, which has been proven to enhance viral entry [25]. Similarly, His655Tyr sub-stitution, which is one of the lineage-defining mutations of the Omicron variant (line-age B.1.529), besides conferring escape potential, is responsible for the preferential us-age of the cathepsin B/L-dependent endosomal entry pathway, thus possibly hamper-ing cell entry and fusogenicity, ultimately attenuating viral pathogenicity [26]. Addi-tionally, we identified Lys417Asn, Asn501Thr, Ala570Val and Ala684Val substitutions, which were shown to enhance binding affinity to the ACE2 cell receptor, hence pro-moting viral infectivity [27,28,29]. Of note, the amino acid at position 501 is replaced by a tyrosine in lineages B.1.1.7, B.1.351 and P.1, while the same Lys417Asn is also present in lineages B.1.529, B.1.351 and P.1, with similar consequences on antibody neutralisa-tion escape and viral infectivity [30,31]. Altogether, 8 of the identified mutations (29%) confer an enhanced immune escape capacity; 6 mutations (21%) increase receptor engagement. 

While some of the escape mutations were rather stable during the infection (i.e., Leu141_Tyr144del, Asn501Thr, Ala570Gly), some other were only temporarily acquired, and were later substituted by either the wild-type (Val635Ala) or by another mutation (Leu242_Leu244del/Ala243_Leu244del). Additionally, some other only emerged during the late phase of infection: some of these arose in Day 189 sample and became stable in the following sequences (reaching a frequency above 90%), while other only appeared in the last sample, with relatively low frequencies (approximately ranging from 70 to 85%). The interruption of the study prevented us to further monitor their evolution. The appearance of the majority of escape mutations early during the infection, hint to an involvement of the monoclonal antibodies administration in their emergence and fixation within the population. The fact that a half of the mutations affecting the spike confer escape potential also supports the hypothesis of an exogenous-humoral-immunity-forced directional selection. Similarly, for what regards infectivity-enhancing mutations, some were stably maintained (Asn501Thr and Ala570Gly), but, for the most part, they emerged later during the infection (Lys417Asn, Val635Gly, His655Tyr, Ala684Val). Of the identified escape mutations, the substitutions of amino acids 417 and 501 could be related to monoclonal antibody administration, as both of them reside in epitopes targeted by Bamlanivimab/Etesevimab and have been proven to confer resistance to neutralisation to both antibodies [32]. Of these, Asn501Gly was already present in Day 132 isolate, but the lack of genomic information regarding the first phase of infection makes it difficult to draw conclusion regarding the role played by monoclonal antibodies in its emergence. On the contrary, Lys417Asn only appeared in Day 218 isolate, suggesting another mechanism responsible for its appearance (i.e., enhanced ACE2 receptor binding, as previously discussed. The appearance of both escape- and infectivity-enhancing mutations throughout the infection suggests a continuous viral adaptation to the host niche. No escape- or infectivity-enhancing mutations were identified in genes coding for other structural proteins.

A large number of mutations (9 of 28, corresponding to 32%) affected ORF1ab, thus involving non-structural proteins, which intervene in various ways during viral replication cycle, including nsp2 (responsible of cellular processed shut-down), nsp3 (papain-like protease), nsp12 (RNA-dependant RNA-polymerase), nsp14 (specifically the N7-Methyltransferase domain), nsp15 (endo-RNase) and nsp16 (2′-O-Ribose-Methyltransferase) [33]. This set of mutations, which, for the most part, was stable in all sequenced samples, may have also contributed to adaptation, hence favouring viral replication in the host. No mutation was detected on the sequence coding for 3C-like protease (3CLpro, or nsp5), which represents Paxlovid molecular target, hence suggesting that treatment with this antiviral did not affect viral evolution. 

## 4. Discussion

Our study describes the dynamics of intra-host viral evolution in the context of a deeply immunocompromised patient, who sustained high-titre replication for a pro-tracted period and was unable to develop a specific humoral immune response capable of counteracting the infection. Several other studies regarding chronic infections in immunocompromised patients suffering from lymphoid malignancies and therefore on anti-CD20 monoclonal antibodies (like Rituximab) maintenance therapies [34,35]. These treatments, by directly targeting B-cells, and, consequently hampering CD4+ T-cells maturation and functionality, ultimately result in a combined depletion as a distinctive hallmark, as observed in our patient, who presented with low B-cell num-ber and consequently diminished CD4+/CD8+ cell ratio. If and to which extent these cellular immunity defects contribute to viral replication and evolutive dynamics is still poorly understood and certainly constitutes breeding ground for further research in this field. This often leads to a treatment-induced long-standing immunosuppression, thus not only protracting the course of SARS-CoV-2 infection, but also exposing pa-tients to recurrent flares following apparent symptoms resolution, reinfections or su-per-infections. This generated a controversy over anti-CD20 antibodies usage for lym-phoma patients during COVID-19 pandemic. Despite data showing an increased inci-dence of SARS-CoV-2 infections in this class of patients [35], to date, Rituximab still represents the best option in addition to first-line chemotherapy treatments to maxim-ise remission duration, which represents the major predictor of tumour aggressiveness for follicular non-Hodgkin lymphomas. For this reason, current recommendations call upon clinicians to carefully evaluate disease burden at diagnosis, long-term benefits of anti-CD20-based treatments and COVID-19 infection-related risks case by case [36].

While in this group of patients reinfections and reactivations are both relatively common events [37], in our case, several reasons argue for a protracted infection started in December 2021, and later reactivated between January and February 2022, rather than a reinfection. First of all, epidemiological records regarding SARS-CoV-2 variants prevalence in Italy support the persistent infection hypothesis: B.1.617.2 prevalence quickly dropped from 99% at the beginning of December 2021 to 20% at the beginning of January 2022, later further decreasing to 0.9% by the end of the month. This trend was mirrored by B.1.1.529 emergence, which rapidly became the predominant variant in Italy, accounting for nearly the totality of recorded cases ever since [38,39,40,41]. For our patient, the first positive sample after clearance at the end of December 2021 dates back to 2 February 2022, but no tests were actually performed in January, making it impossible to precisely determine for how long our patient was COVID-free and, consequently, when reactivation or reinfection events might have occurred. Despite this, a putative reinfection between January and February would have more probably involved B.1.1.529 lineage, rather than B.1.617.2, thus making the reactivation hypothesis more probable. Furthermore, the patient did not report a close contact with a positive person prior to February. In conclusion, although genomic data regarding the early phase of infection is lacking, these information hint that the original B.1.617.2 AY.122 virus underwent a reactivation and then followed a divergent evolutionary pathway within the infected host, which at first allowed the differentiation of diverse subpopulations, with a subsequent selection and further evolution of one of these subpopulations. These dynamics argue for viral evolution and against reinfection. 

In particular, whole genome sequencing disclosed the longitudinal emergence of different and genetically distinct (although related) viral populations, which alternated during the course of infection. It could be speculated that the cyclic emergence and disappearance of distinct genotypes may derive from a dynamic interplay between viral replication and host factors, which led to the emergence and, in some cases, fixation, of mutations responsible of immune escape or enhanced viral infectivity, leading to an overall host adaptation. Moreover, the appearance of 9 escape mutations on the spike also suggests a role of the passive immunity induced by monoclonal antibody administration in the early phase of infection in viral evolution, which induced site-specific forced mutagenesis in immune-relevant epitopes of the spike glycoprotein, and thus exerted a selective pressure directed toward immune escape. Other mutations affected key non-structural proteins involved in replication cycle, maybe enforcing adaptation to the host cellular environment too. 

This study not only describes the clinical course of a complex case of persistent SARS-CoV-2 infection in an oncological and immunosuppressed patient, but also analyses intra-host viral evolution over the course of the infection. Under this point of view, it documents the appearance of new mutations, some classified as escape mutations, some other as infectivity-enhancing mutations, which often seemed to evolve by convergent evolution in other circulating variants, as well as shedding light into a peculiar and dynamically sophisticated evolutionary pattern, which has shown the accumulation of a great number of mutations, some of which were persistent, while some other were cyclically acquired and lost, either replaced by the wild-type or by another mutation, in a fluctuating fashion. 

Although our study describes a single individual, whose underlying conditions, clinical course and laboratory findings may not be broadly generalizable to other immunocompromised subpopulations, this case highlights how immune defects lead to uncontrolled SARS-CoV-2 infection, whose genetic plasticity is able to shape a genetically structured mutational landscape within a single infected individual in response to an externally imposed selective pressure (in this case represented by monoclonal antibodies administration). 

This study may also contribute to elucidate relevant aspects of SARS-CoV-2 evolution and their potential repercussions on general prevention and containment strategies, as well as on genomic surveillance initiatives. In fact, some of the identified mutations, which have been associated with increased transmissibility and/or increased resistance to antibody neutralization, are characteristic of some of the viral variants emerged in the last two years. These findings support the hypothesis that people with protracted infection may have been the origin of some of the highly mutated variants that have been identified to date. In this perspective, immunocompromised subjects would seem to provide fertile ground for the genetic evolution of SARS-CoV-2, and, although representing the minority of the infected population, their potential to harbour new viral variants should not be neglected, in view of their theoretical augmented transmissibility and immune escape capacity, as already speculated for the emergence of lineage B.1.1.7 in the United Kingdom [14].

## Figures and Tables

**Table 1:** Overview of the molecular routine testing for SARS-CoV-2 on nasopharyngeal swabs collected between December 2021 and July 2022. All samples were tested using Xpert Xpress SARS-CoV-2 (Cepheid, Sunnyvale, CA, USA). All samples from Day 132 were sequenced as part of this study.

Sample	Date	N2 ^1^ Gene	E ^2^ Gene
Day 1	3 December 2021 *	21	22
Day 17	20 December 2021	23	24
Day 25	28 December 2021	N/D ^3^	N/D
Day 61	2 February 2022	22	24
Day 83	24 February 2022	18	19
Day 99	12 March 2022	20	19
Day 117	30 March 2022	21	23
Day 132	14 April 2022	24	23
Day 144	26 April 2022	20	23
Day 152	4 May 2022	15	17
Day 165	17 May 2022	16	19
Day 176	28 May 2022	19	17
Day 189	10 June 2022	19	19
Day 200	21 June 2022	21	24
Day 218	9 July 2022	20	21

^1^ N = nucleocapsid; ^2^ E = envelope; ^3^ N/D = not detected. * indicates that on the same day the patient received Bamlanivimab/Etesevimab.

**Table 2:** Overview of the serological testing performed from December 2021 to June 2022. Anti-S IgG were determined with a commercial CLIA-based kit, LIAISON SARS-CoV-2 trimeric-S (DiaSorin, Vicenza, Italy), while anti-N IgG were measured with Abbott SARS-CoV-2 anti-NP IgG (Abbott Core Laboratory Systems, Lake Forest, IL, USA).

Sample	Date	Anti-S ^1^ IgG (BAU ^3^/mL)	Anti-NP ^2^ IgG (AU ^4^/mL)
Day 24	27 December 2022	1500	N/D ^5^
Day 111	23 March 2022	1300	N/D
Day 152	4 May 2022	333	N/D
Day 176	28 May 2022	199	N/D
Day 200	21 June 2022	194	N/D

^1^ S = spike, ^2^ NP = nucleocapsid protein; ^3^ BAU = binding antibody unit; ^4^ AU = arbitrary unit; ^5^ N/D = not detected.

**Table 3:** Overview of the intra-host mutations developed during a prolonged infection in an immunocompromised patient compared to a reference B.1.617.2 AY.122 consensus sequence (Access: OW998398.1). For each identified mutation affected protein (and domain) are shown in order to better understand its potential role on viral biology and consequently on adaptation to the host. Mutations with a putative role in viral adaptation are more deeply described in the main text. Percentage mutation frequencies show a peculiar fluctuating pattern, in which newly identified mutations tend to be replaced either by the wild-type or by another mutation; this enabled us to differentiate three distinct but related subpopulations: subpopulation 1 (Day 132, Day 152 and Day 176 samples), subpopulation 2 (Day 144 and Day 165 samples) and subpopulation 3 (Day 189, Day 200 and Day 218 samples).

Gene	Genome Position	Nucleotide Change	Affected Protein (Domain)	Amino Acid Change	Mutation Frequency (%)							
					Day 132	Day 144	Day 152	Day 165	Day 176	Day 189	Day 200	Day 218
ORF ^1^ 1ab	1363	T>A	nsp2 ^5^	Val366=		96		98.9		99.6	99.2	99.3
	6294	T>C	nsp3	Ile2010Thr								75.4
	7654	A>G	nsp3	Thr2463=	98.6	98.2	79.1	99.4	95	99.8	99.9	99.8
	9936	C>T	nsp4	Thr3224Ile								80.8
	10038	C>A	nsp4	Thr3258Asn	99.2	99.5	99.6	99.7	99.8	99.7	99.7	99.9
	13451	G>T	nsp12	Ala4396Ser	98.5	98.5	76.6	99.7	95.8	99.7	99.7	99.6
	18337	G>T	nsp14	Ala6025Ser						74.8	71.9	91.4
	20275	G>T	nsp15	Asp6671Tyr	99.7	99.4	99.9	99.6	99.9	100	99.9	99.8
	20871	T>C	nsp16	Gly6869=	98.9	99.4	99.8	99.4	99.8	99.2	99.6	99.6
S ^2^	21764	A>G	Spike (NTD ^6^)	Ile68Val						81.7	88.8	90.1
	21956	G>C	Spike (NTD)	Glu132Gln								81.4
	21981	12 nt ^4^ del	Spike (NTD)	Leu141_Tyr144del	99.9	100	100	94.2	99	100	100	97.9
	22280	9 nt del	Spike (NTD)	Leu242_Leu244del		81.8		81.7		94.2	99.4	97.4
	22288	6 nt del	Spike (NTD)	Ala243_Leu244del			100		99.3			
	22813	G>T	Spike (RBD ^7^)	Lys417Asn								84.4
	23064	A>C	Spike (RBD)	Asn501Thr	99.8	99.5	99.7	100	99.9	100	99.8	100
	23271	C>G	Spike (S2 ^8^)	Ala570Gly	99.1	99.3	99.8	99.8	99.4	99.7	99.7	99.9
	23466	T>C	Spike (S2)	Val635Ala	97.8		99.9		99.9			
		T>G	Spike (S2)	Val635Gly						92.6	98.7	97.4
	23525	C>T	Spike (S2)	His655Tyr						93.4	99.1	98.8
	23613	C>T	Spike (S2)	Ala684Val								71.4
	24871	T>A	Spike (S2)	Phe1103Leu								82.9
	25019	G>T	Spike (S2)	Asp1153Tyr						99.2	99.3	98.9
ORF7a	27526	C>G	ORF7a	Pro45Ala	98.9	99.7	100	99.7	100	99.3	99.9	100
	27680	T>C	ORF7a	Leu96Pro	98.9	99	99.4	99.6	99.6	99.5	99.6	99.9
N ^3^	28474	T>C	Nucleocapsid	Pro67=	98.1	98.8	99.6	99.1	98.9	98.8	98.6	98.8
	28557	G>A	Nucleocapsid	Arg95His	99.5	98.9	99.5	99	99.8	98.8	97.9	99.9
ORF10	29535	C>T			88.6	97.4	71.9	99.8	100	100	99.7	99.9

^1^ ORF = open reading frame; ^2^ S = spike; ^3^ N = nucleocapsid; ^4^ nt = nucleotide; ^5^ nsp = non-structural protein; ^6^ NTD = N-terminal domain; ^7^ RBD = receptor binding domain; ^8^ S2 = sub-unit 2. The “=” symbol indicates a synonym mutation, while “del” indicates a nucleotide deletion.

## Data Availability

All sample sequences are available through GISAID. Their accession numbers are listed below: EPI_ISL_15528152 (Day 132), EPI_ISL_15528328 (Day 144), EPI_ISL_15528329 (Day 152), EPI_ISL_15528330 (Day 165), EPI_ISL_15528331 (Day 176), EPI_ISL_15528332 (Day 189), EPI_ISL_15528333 (Day 200), EPI_ISL_15528334 (Day 218).
